# Enhanced Ageing Performance of Sulfonic Acid-Grafted Pt/C Catalysts

**DOI:** 10.3390/mi13111825

**Published:** 2022-10-26

**Authors:** Yuzhen Xia, Hangwei Lei, Chuanfu Sun, Xiaohao Wen, Zichen Wang, Guilin Hu, Baizeng Fang

**Affiliations:** 1 School of Mechanical and Energy Engineering, Zhejiang University of Science and Technology, Hangzhou 310023, China; 2 Department of Chemical and Biological Engineering, University of British Columbia, Vancouver, BC V6T 1Z3, Canada

**Keywords:** Pt electrocatalyst, sulfonic acid group, chemical functionalization, adsorption energy, electrochemical stability, density functional theory

## Abstract

Chemical functionalization of carbon support for Pt catalysts is a promising way to enhance the performance of catalysts. In this study, Pt/C catalysts grafted with various amounts of phenylsulfonic acid groups were prepared under mild conditions. The influence of sulfonic acid groups on the physiochemical characteristics and electrochemical activities of the modified catalysts were studied using X-ray diffraction, X-ray photoelectron spectroscopy, a transmission electron microscope, and cyclic voltammetry (CV). The presence of the chemical groups enhanced the hydrogen adsorption onto/desorption off the Pt surface during the CV cycling. In contrast, the hydrogen peaks of the grafted catalysts increased after 500 CV cycles, especially for Pt (111) facets. The highest electrochemical surface area (ECSA) after the aging test was obtained for the catalyst with 18.0 wt.% graft, which was ca. 87.3% higher than that of the non-functionalized Pt catalyst. In the density functional theory (DFT) calculation, it was proven that SO_3_H adsorption on the crystalline was beneficial for Pt stability. The adsorption energy and bond distance of the adsorbed SO_3_H on Pt (110), (100), and (111) surfaces were calculated. All the stable configurations were obtained when O from S-O single bond or S was bound to the Pt surface, with the adsorption energy following the trend of (111)_F_ > (100)_H_ > (110)_H_. This result was consistent with the ECSA experiment, which explained the high electrochemical stability of the sulfonic acid groups-grafted Pt/C catalyst.

## 1. Introduction

In low-temperature fuel cells, the catalyst plays a critical role in reducing the reaction activation barrier. Platinum (Pt) and Pt-based alloys are the most commonly used catalysts due to their high catalytic activity, chemical stability, high exchange current density (i_0_), and superior work function [1,2,3,4,5]. However, the global scarcity of Pt contributes to the high price of fuel cells. The poor stability of Pt nanoparticles also casts a shadow on the commercial application of fuel cells and other oxygen reduction reaction (ORR)—involved energy conversion devices [6]. A common solution is to disperse small particles of platinum or platinum-based alloys on the surface of support materials [7,8,9]. Materials with good electrical conductivity, which can facilitate fast charge transport, are also necessary for high performance [10,11]. Carbon materials, with a high surface area, are generally used as supports to disperse noble metal catalysts and facilitate the access of reactants to catalytic sites and the removal of reaction products [12]. The properties of carbon supports have been found to have a strong influence on the performance of metal-supported catalysts due to their morphology, size distribution, and stability [13,14]. The research aiming to improve Pt mass activity for the ORR requires the optimization of both the specific activity and the electrochemically active surface area (ECSA) [15]. It has been reported that increasing the number of surface oxygen groups on the support increases ECSA values and ORR activity [16,17]. The surface graft of the support also influences the hydrophobic/hydrophilic character of the materials. Therefore, activating their surfaces may be necessary to improve catalyst utilization [18,19,20]. Specifically, electrocatalytic activity and durability could be varied significantly depending on the surface defects [21,22].

Sulfonation of carbon, which provides support materials with ionic and electronic conductivity, has been thought to be a promising way to enhance the performance of catalysts [23,24]. Catalysts supported on sulfonated carbon have been prepared by various modification methods, and their electrochemical performance in fuel cells has been investigated [25,26,27,28,29,30,31,32,33]. Kim et al. [25] functionalized Ketjen Black EC 300J (KB) with a sulfonated polymer by adding a certain amount of 2,2-azobisisobutyronitrile (AIBN) to the mixture of KB and styrene monomer at 65 °C for radical polymerization of styrene, which was washed with tetrahydrofuran for the sulfonation reaction of polystyrene. Du et al. [26] prepared a sulfonated Pt/CNT catalyst by thermal decomposition of (NH_4_)_2_SO_4_ and in situ radical polymerization of 4-styrenesulfonate. Sun et al. [27] grafted functionalized benzenesulfonic groups onto the surface of multiwalled carbon nanotube (MWCNT)-supported Pd catalysts by in situ radical polymerization of 4-styrenesulfonate and isoamyl nitrite in the presence of 96% H_2_SO_4_, (NH_4_)_2_S_2_O_8_ and AIBN at 80 °C. Sun et al. [28] prepared sulfonated ordered mesoporous carbon (OMC) by in situ radical polymerization of 4-styrenesulfonate and isoamylnitrite at 70 °C. Weissmann et al. [31] prepared hydrophilic phenylsulfonic acid group-grafted carbon supports via diazonium ion reduction. Without the addition of a strong acid or high temperature, 4-aminophenylsulfonic acid was transformed into diazonium in the presence of NaNO_2_. A higher ECSA and enhanced performance in methanol electrooxidation were shown. It has been widely concluded from previous reports that the electronic interaction between the surface groups and Pt atoms could favor electron transfer from catalytic sites to the conductive carbon electrode, or chemical groups of the support can act as anchors for metal particles, preventing their agglomeration.

The electrochemical activity of Pt catalysts could be quantitatively determined by measuring hydrogen adsorption/desorption in water solution by cyclic voltammetry (CV). ECSA calculation assumes that H adsorption/desorption charge density on polycrystalline Pt is 210 μC∙cm^−2^, derived from equal distribution of the three low index planes (111), (110) and (100) of Pt [24]. The adsorption properties of hydrogen on their surface could be studied by density functional theory (DFT) [34,35]. Considering the influence of chemical groups on the catalytic performances of Pt, Ungerer et al. [36] studied the adsorption of S, SO, and SO_3_ on the Pt (001), (011), and (111) surfaces.

The use of supported electrocatalysts is a proven and efficient strategy to reduce Pt usage and improve the electrocatalytic activity of Pt-based electrocatalysts. However, electrochemical oxidation of carbon support after an extended period of operation results in the agglomeration and shedding off the supported Pt and eventually leads to a loss in the electrocatalytic activity and fuel cell performance. The sulfonation of carbon via diazonium ion reduction offers a simple method for the preparation of efficient support materials without the need for heat treatment at an elevated temperature. The enhanced properties have usually been attributed to the electronic interaction between the surface groups and Pt atoms, while the reason for high electrochemical stability has not been explored. In this work, sulfonated Pt catalysts grafted with phenylsulfonic acid groups were prepared by the diazonimum synthesis route under mild conditions. The effects of the graft ratio on the electrocatalytic performance and stabilities were studied in a three-electrode system. DFT calculations were used to predict the behavior of sulfonic acid on the Pt (110), (100), and (111) surfaces for further understanding of the aging mechanism of the Pt catalysts.

## 2. Methods and Models

### 2.1. Experiments

The deposition of platinum particles onto Vulcan^®^ XC-72R (Tianjin Avison Chemical Technology Co., Ltd., Tianjin, China) was carried out by the polyol method. Functionalization with phenylsulfonic acid was carried out via diazonium ion reduction, a method developed by Weissmann et al. [31]. A suspension containing 4-aminobenzene sulfonic acid (AR, Shanghai Zhangyu Chemical Co., Ltd., Shanghai, China), 10 mL of 0.5 M HCl, and 60 mg of Pt catalyst was stirred for 0.5 h. Then, NaNO_2_, up to a concentration twice that of 4-aminobenzene sulfonic acid, was dissolved in 15 mL of water and added dropwise to the suspension. This mixture was stirred at 5 °C for 24 h and then filtered and rinsed thoroughly with deionized water. The washed residue was then placed in an oven at 60 °C for 48 h. The weight of 4-aminobenzene sulfonic acid were accounting for 5.8, 11.6, 18.0, and 23.3 wt.% of the mass of the catalyst, with the corresponding modified catalysts named Pt/C-S1, S2, S3, and S4, respectively.

X-ray diffraction (XRD) analysis of the as-prepared Pt catalyst before and after the sulfonation was performed with an Ultima IV X-ray diffractometer from Rigaku Corporation using Cu Ka radiation and operating at 45 kV and 40 mA. X-ray photoelectron spectroscopy (XPS) spectra were acquired by using a Thermo Scientific K-Alpha spectrometer (Thermo Scientific Co., Ltd., Waltham, MA, USA) equipped with a monochromatic Al X-ray source (Al Kα, 1.4866 keV). The dispersion of the platinum particles was identified from the transmission electron microscope (TEM) images obtained using a JEM-1200EX TEM (Japan Electronics Co., Ltd., Tokyo, Japan).

The ECSA of catalysts was investigated in a three-electrode system using a potentiostat CHI600E (Shanghai Chinstruments Co., Ltd., Shanghai, China). In the system, a catalyst-coated glassy carbon electrode (working electrode), a Pt coil (counter electrode), and a saturated Ag/AgCl electrode (reference electrode) were put into 0.5 M HClO_4_ solution at 25 °C. The working electrode was prepared by dispersing 5 mg of catalyst in 0.5 mL of ethanol and 2 mL of dilute Nafion solution (0.0125 wt.% in water) after performing ultrasonic treatment for 30 min and by pipetting 10 μL of the suspension onto the glassy carbon electrode (ϕ = 5 mm). The electrochemical properties of the catalysts were determined by performing cyclic voltammetry (CV) from 0 to 1.0 V (NHE) with a scan rate of 50 mV/s.

### 2.2. Computational Methods

DFT calculations were carried out using CASTEP code in Materials Studio with generalized gradient approximation (GGA) and PBE exchange–correlation functional proposed by Perdew, Burke, and Ernzerhof. Plane waves were included with a recommended cutoff of 380 eV. The fcc Pt lattice constant was 3.924 Å, equal to the experimental value [37]. The surface of Pt (100), (110), and (111) was modeled by a p (3 × 3) supercell comprised of a three-layer slab, where Pt atoms in the top and second layer were allowed to relax whereas those in the bottom layer were fixed at the calculated bulk positions. Each slab has a vacuum space of 15 Å. Monkhorst-Pack k-point mesh of 2 × 2 × 1 was used in the integrations in reciprocal space.

The average adsorption energy (E_ads_) per adsorbate molecule (SO_3_H) adsorbed onto the Pt surface was calculated with the following equation [36]:

(1) $$E_{\mathrm{abs}}=\frac{1}{N_{{\mathrm{SO}}_{3}H}}\left[E_{\mathrm{Pt},r}^{N_{{\mathrm{SO}}_{3}H}\neq 0}-\left(E_{\mathrm{Pt},r}^{N_{{\mathrm{SO}}_{3}H}=0}+N_{{\mathrm{SO}}_{3}H}E_{{\mathrm{SO}}_{3}H}\right)\right]$$

where $N_{{\mathrm{SO}}_{3}H}$ was the number of adsorbed SO_3_H molecules, $E_{\mathrm{Pt}}^{N_{{\mathrm{SO}}_{3}H}\neq 0}$ was the energy of the Pt slab with adsorbed SO_3_H molecules, $E_{\mathrm{Pt}}^{N_{{\mathrm{SO}}_{3}H}=0}$ was the energy of the clean Pt surface, and $E_{{\mathrm{SO}}_{3}H}$ was the energy of the isolated SO_3_H molecule after relaxation.

## 3. Results and Discussion

The sulfonated Pt catalysts were prepared by applying a 4-phenylsulfonic acid layer onto the surface of a Vulcan in an aqueous medium by in situ generated diazonium cations. The solution of Pt catalyst and 4-aminobenzene sulfonic acid was vigorously stirred to ensure uniform mixing. The addition of NaNO_2_ resulted in the transformation of an amine into diazonium despite the release of nitrogen dioxide and nitric oxide gas [31].

**Figure 1:**
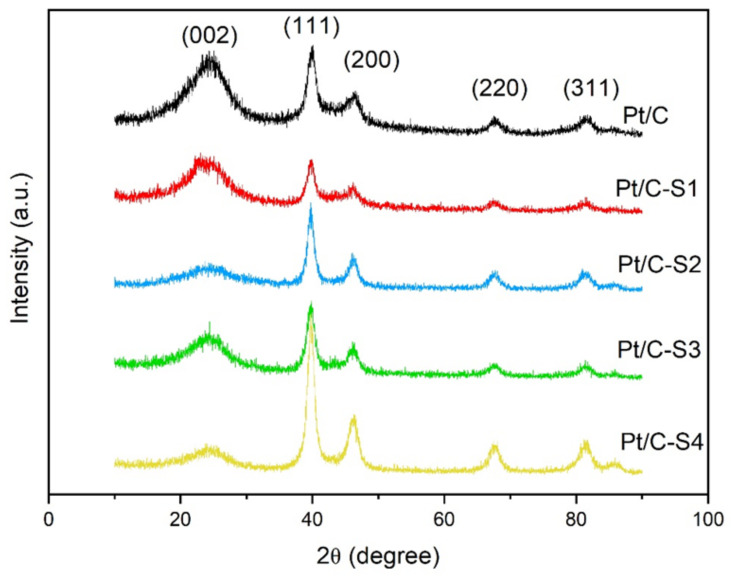
XRD patterns of the Pt/C and sulfonated catalysts.

The XRD patterns of the Pt catalysts before and after the sulfonation are shown in Figure 1. Each catalyst exhibited hexagonal graphite (002) structures and face-centered cubic structures of Pt (111), (200), (220), and (311). No obvious peak position shift is found after the sulfonation, indicating that the diazonium ion reduction of the Pt/C catalyst does not significantly affect the crystal structure of Pt in the catalysts [26]. The broader and weaker (002) peak for sulfonated Pt catalysts may be attributed to the successful coating by sulfonic acid [38]. The average particle sizes of the catalysts could be calculated from Pt (220) diffraction peaks at 2θ of ca. 67.5° using Scherrer’s equation because it was isolated from the diffraction peaks of carbon support [39,40]:

(2) $$L=0.89\lambda /\left(B_{2\theta }{\mathrm{Cos}\theta }_{B}\right)$$

where L was the average particle size in nm, λ was the wavelength of the X-rays (1.54056 Å), θ was the angle at the maximum of the peak, and B_2θ_ was the width of the peak at half-height.

When the value of the Pt/C catalyst was 5.0 nm, the particle size of the Pt/C-S series was ca. 5.1–5.4 nm. The graft amount had little effect on the Pt particle distribution.

**Figure 2:**
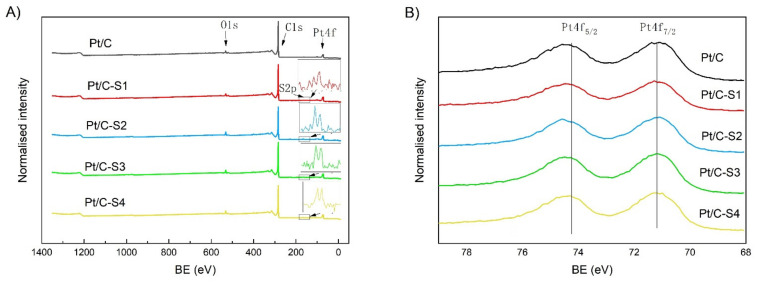
The XPS survey spectra of the Pt/C and sulfonated catalysts (**A**) and high-resolution spectra for platinum Pt 4f component (**B**).

XPS was carried out to detect the elements present and determine the chemical composition on the near-surface of 2~10 nm. Elemental analysis extracted from the XPS measurements verified the presence of sulfur after the sulfonation functionalization, as shown in Figure 2A, with a percentage of S element estimated to be ca. 2.0, 2.5, 2.7, and 3.0 wt.% of the catalysts, respectively. In Figure 2B, the platinum component shows the typical Pt(0) 4f 5/2 and 4f 7/2 peaks (74.6 and 71.3 eV, respectively), confirming the amount of metallic platinum around 17.0 wt%.

**Figure 3:**
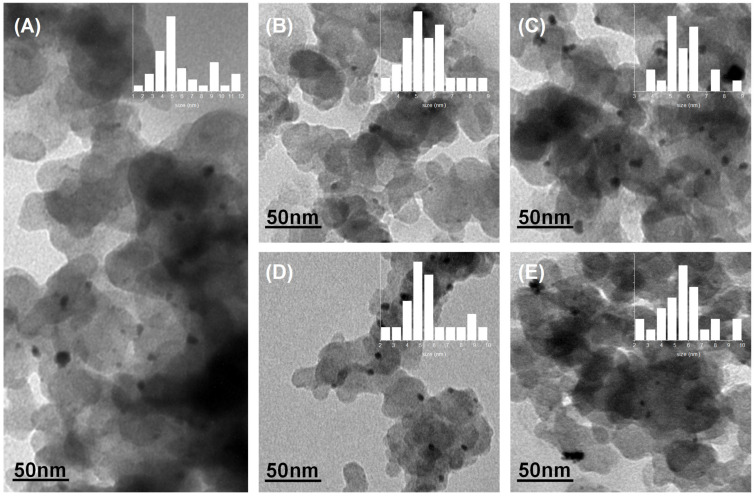
TEM images of the Pt/C (**A**) and sulfonated catalysts: Pt/C-S1 (**B**), Pt/C-S2 (**C**), Pt/C-S3 (**D**) and Pt/C-S4 (**E**).

TEM images of the Pt/C and Pt/C-S catalysts grafted with various amounts of graft are shown in Figure 3. The platinum particles had a relatively random dispersion on the carbon support. The size in Figure 3A–E was found to be very close, around 5.0 nm, and all highly uniform. This observation was consistent with the XRD results, indicating that the sulfonation of the Pt/C catalyst via diazonium ion reduction did not cause significant agglomeration of platinum particles. The chemical groups, covalently bonded to Vulcan, would provide anchoring sites that enabled the homogeneous distribution of the Pt nanoparticles during the following aging tests.

**Figure 4:**
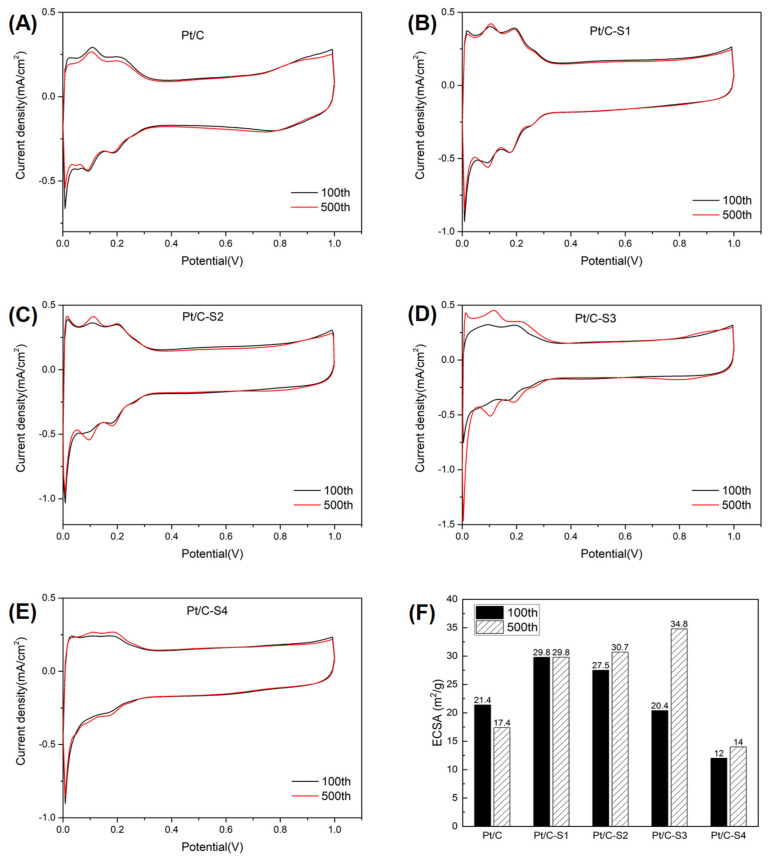
CV curves (**A**–**E**) and obtained ECSA (**F**) of the Pt/C and sulfonated catalysts after 100 and 500 cycles.

The electrochemical activities of the catalysts prepared with and without the sulfonic acid grafts were explored by performing repetitive CV tests with cycling the potential in the region of 0.0–1.0 V (NHE) for up to 500 cycles. Due to the impurity of the three-electrode system, the CV curves after 100 cycles were used to estimate the initial electrochemical activities of the Pt catalysts and compared with those obtained after 500 cycles, as shown in Figure 4.

All the catalysts depicted well-defined CV curves, showing Pt-O reduction and hydrogen desorption peaks from the Pt surface in the cathodic scan and adsorption peaks of Pt-OH/Pt-O formation in the anodic scan. As to the Pt/C catalyst, the intensity of hydrogen adsorption/desorption peaks decreased after 500 cycles, as shown in Figure 4A, with the peaks shifting to a lower potential, suggesting an alteration of catalyst surface [33]. Three well-defined peaks with opposite currents were observed in the CV curves of Pt/C-S1, S2, and S3 catalysts, shown in Figure 4B–D, indicating H desorption on the surface of Pt with different crystalline forms. Meanwhile, higher peaks were shown after 500 cycles, which concluded higher durability of the Pt/C-S series catalysts. Compared to the dissolution/redeposition of Pt that occurred in the Pt/C catalyst, less loss of high energy facets and lower amount of lower energy facets were concluded because of the sulfonic acid graft [41].

ECSA (m^2^ g^−1^) could be estimated by the integration of hydrogen adsorption or desorption peak after the double layer charging current in the potential range of 0.05–0.45 V (NHE) subtracted, based on the assumption of a charge value of 0.210 mC cm^−2^ for the oxidation of a monolayer of adsorbed hydrogen on polycrystalline Pt [42].

ECSA = Q_H_/0.21 · L_Pt_ (3)

where Q_H_ was the average of the estimated adsorption and desorption charge, mC cm^−2^, and L_Pt_ was the Pt loading in the electrode disk.

For the early period of repetitive CV, the value of ECSA increased slightly after sulfonic acid grafting. However, it began to decrease as the graft ratio was above 18.0%. The initial ECSA of the Pt/C-S3 and S4 was lower than that of Pt/C. The hydrophilicity of sulfonic acid graft might hinder the hydrogen adsorption/desorption on Pt surface. The value of ECSA after 500 cycles also increased after sulfonation. It might be attributed that the adsorption energy of SO_3_H onto Pt surface was higher than that of hydrogen, which therefore restricted the hydrogen adsorption/desorption on the Pt surface initially. As CV cycles were processed, the hydrophilicity of sulfonic acid graft resulted in water adsorption on the surface, and therefore more the Pt surface was free for hydrogen adsorption/desorption. The highest ECSA was obtained by sulfonating with 18.0% graft, which was 87.3% larger than that of Pt/C catalysts.

**Figure 5:**
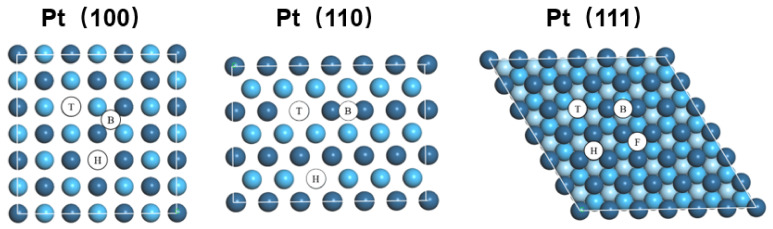
Adsorption sites of the Pt (001), (011), and (111) surfaces indicated as top (T), bridge (B), hexagonal close packed (H), and face-cubic centered (F), where Pt atoms are blue in color, while the second layer and third layer are displayed in a lighter color and the lightest color.

For further understanding of the influence of sulfonic graft, the adsorption of SO_3_H on the Pt (110), (100), and (111) surfaces were studied by DFT technology. The adsorption sites of Pt (100), (110), and (111) are shown in Figure 5. Both Pt (100) and (110) had three adsorption sites, indicated by top (T), bridge (B), and hexagonal close-packed (H), while Pt (111) has four sites indicated by top (T), bridge (B), hexagonal close-packed (H), and face-cubic centered (F).

**Figure 6:**
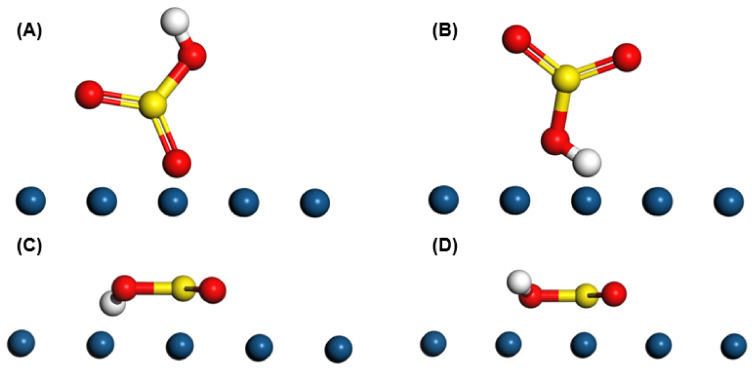
Four modes of SO_3_H adsorption: (**A**) SO_3_ plane was parallel to Pt surface, with H atom interacted, (**B**) SO_3_ plane was parallel to Pt surface, with H atom directed away, (**C**) SO_3_ plane was perpendicular to Pt surface, with H atom interacted, (**D**) SO_3_ plane was perpendicular to Pt surface, with H atom di-rected away.

The model of the sulfonic acid group was optimized in Materials Studio, where four atoms of S, O, O, O, were in a plane, as shown in Figure 6. The golden ball represented the S molecule, the red ball represented the O molecule, and the white ball represented the H molecule. The bond length of S=O, S-O, and O-H were 1.780, 1.780, and 1.112 Å, respectively. The four possible modes of SO_3_H adsorption considered in this work include (i) SO_3_ plane parallel to Pt surface, with the H atom interacting, (ii) SO_3_ plane parallel to Pt surface, with the H atom directed away, (iii) SO_3_ plane perpendicular to Pt surface, with the H atom interacting, and (iv) SO_3_ plane perpendicular to Pt surface, with the H atom directed away.

**Figure 7:**
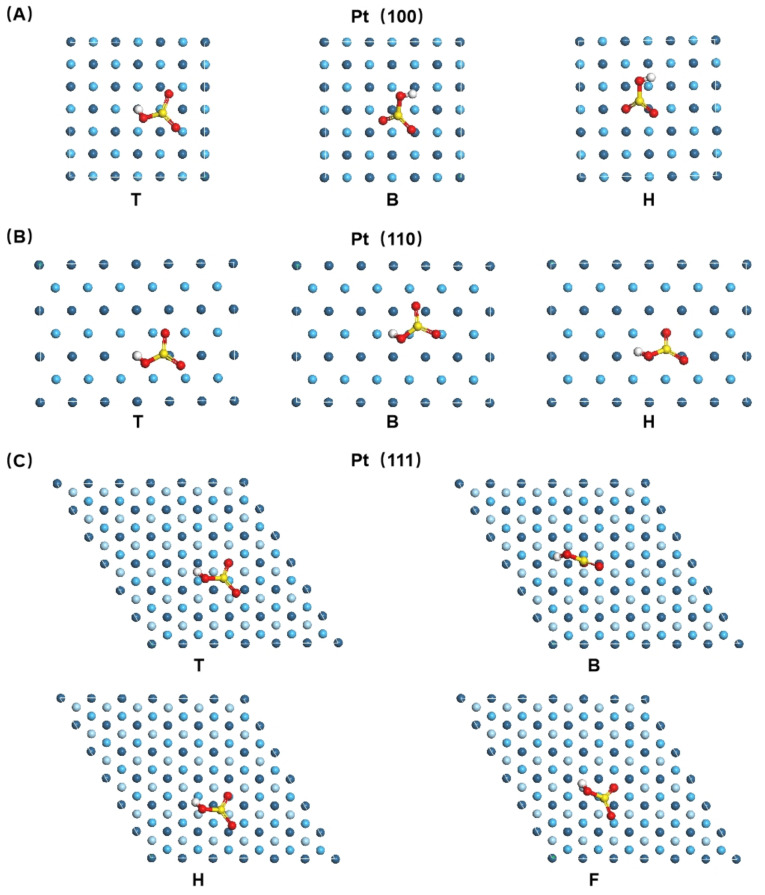
Adsorption of sulfonic acid group at different sites on Pt (001), (011), and (111) surfaces.

The influence of the sulfonated group on the catalytic properties of Pt was studied by DFT calculation. Four possible modes of SO_3_H adsorption were considered in this work. The most stable configurations of SO_3_H on Pt (100), (110), and (111) surfaces are shown in Figure 7, with the adsorption energies and bond distances listed in Table 1. Three stable SO_3_H adsorption configurations for the Pt (100) surface on T, B, H sites, respectively, as shown in Figure 7A, were obtained when the O in the S-O was bound to the Pt surface. In contrast, the highest adsorption energy was achieved on the H site, followed by the B site and T site. On the Pt (110) surface, three stable adsorption configurations were observed in Figure 7B, one S-bound and two O-bound, in which O was from the single bond of S-O. The most stable was the H site configuration, with E_ads_ at −3.63 eV. The (111) surface achieved four stable adsorption configurations, all of which were O-bound configurations, as shown in Figure 7C. The highest adsorption energy, −6.14 eV, was achieved on (111)_F_, which was higher than those on (100)_H_ and (110)_H_.

## 4. Conclusions

The physical properties of Pt/C catalysts grafted with 4-phenylsulfonic acid were first studied by XRD and TEM. No obvious aggregation of the Pt particles was observed due to the sulfonation. Their electrochemical activities were studied in a three-electrode system by cyclic voltammograms. The initial intensity of the hydrogen adsorption/desorption peak first increased and then decreased due to the sulfonic acid graft. The presence of sulfonic acid resulted in water adsorption in the Pt catalyst, therefore hindering the hydrogen accessibility to the surface of Pt. After 500 cycles, remarkably higher electrochemical activity was depicted in the sulfonated catalysts. Three distinct peaks were observed in the CV curves, relating to hydrogen adsorption on (110), (100), and (111), respectively. The catalyst with 18.0 wt% graft exhibited the highest ECSA. DFT calculation proved that the adsorption energy of sulfonic acid groups on different platinum crystals showed a trend of (111)_F_ > (100)_H_ > (110)_H_. This result was consistent with the experiment, where the hydrogen adsorption peak, especially on (111), was obviously enhanced after the graft of sulfonic acid. Therefore, it could be concluded that the introduction of the graft to the Pt/C catalysts by sulfonation could protect the crystalline form of Pt catalysts and improve their electrochemical activities and stability.

## Figures and Tables

**Table 1:** Adsorption energies (E_ads_), bond distances (d) of the adsorbed sulfonic acid group on the Pt (001), (011) and (111) surfaces.

	Adsorption Site	E_ads_ (eV)	d (Å)
Pt(100)	T	−3.96	2.025 (O-Pt)
	B	−5.40	1.956 (O-Pt)
	H	−5.56	1.990 (O-Pt)
Pt(110)	T	−3.59	2.160 (O-Pt)
	B	−3.44	2.078 (O-Pt)
	H	−3.63	2.228 (O-Pt)
Pt(111)	T	−2.64	2.193 (O-Pt)
	B	−2.07	2.171 (O-Pt)
	H	−4.67	2.075 (O-Pt)
	F	−6.14	2.073 (O-Pt)

## Data Availability

Data will be available upon request from the corresponding authors.
